# Ammonio Methacrylate Copolymer (Type B)-Diltiazem Interactions in Solid Dispersions and *Microsponge* Drug-Delivery Systems

**DOI:** 10.3390/polym14102125

**Published:** 2022-05-23

**Authors:** Iliyan Kolev, Nadezhda Ivanova, Tanya Topouzova-Hristova, Tanya Dimova, Pavlina Koseva, Ivalina Vasileva, Sonya Ivanova, Anton Apostolov, Gergana Alexieva, Atanas Tzonev, Vesselin Strashilov

**Affiliations:** 1 Department of Pharmaceutical Chemistry, Faculty of Pharmacy, Medical University “Prof. Dr. Paraskev Stoyanov”–Varna, 84 “Tzar Osvoboditel” Blvd., 9000 Varna, Bulgaria; tania.dimova@mu-varna.bg (T.D.); pavlina.koseva@mu-varna.bg (P.K.); ivalina.vasileva@mu-varna.bg (I.V.); sonya.j.ivanova@gmail.com (S.I.); 2 Department of Pharmaceutical Technologies, Faculty of Pharmacy, Medical University “Prof. Dr. Paraskev Stoyanov”–Varna, 84 “Tzar Osvoboditel” Blvd., 9000 Varna, Bulgaria; 3 Department of Cytology, Histology and Embryology, Faculty of Biology, Sofia University “St. Kl. Ohridski”, 8 Dragan Tzankov Str., 1164 Sofia, Bulgaria; topouzova@biofac.uni-sofia.bg; 4 Laboratory on Structure and Properties of Polymers, Faculty of Chemistry and Pharmacy, Sofia University “St. Kl. Ohridski”, 1 J. Bourchier Blvd., 1164 Sofia, Bulgaria; apostolov@chem.uni-sofia.bg; 5 Department of General Physics, Faculty of Physics, Sofia University “St. Kl. Ohridski”, 5 J. Bourchier Blvd., 1164 Sofia, Bulgaria; gerry@phys.uni-sofia.bg; 6 Department of Condensed Matter Physics and Microelectronics, Faculty of Physics, Sofia University “St. Kl. Ohridski”, 5 J. Bourchier Blvd., 1164 Sofia, Bulgaria; a_tzonev@phys.uni-sofia.bg (A.T.); amigdaline@yahoo.com (V.S.)

**Keywords:** diltiazem, Eudragit RS, *microsponge* drug delivery, solid dispersions

## Abstract

This paper presents a complex analytical study on the distribution, solubility, amorphization, and compatibility of diltiazem within the composition of Eudragit RS 100-based particles of microspongeous type. For this purpose, a methodology combining attenuated total reflectance Fourier transform infrared (ATR-FTIR) absorption spectroscopy, differential scanning calorimetry (DSC), scanning electron microscopy with energy-dispersive X-ray microanalysis (SEM-EDX), and in vitro dissolution study is proposed. The correct interpretation of the FTIR and drug-dissolution results was guaranteed by the implementation of two contrasting reference models: physical drug–polymer mixtures and casting-obtained, molecularly dispersed drug–polymer composites (solid dispersions). The spectral behavior of the drug–polymer composites in the carbonyl frequency (νCO) region was used as a quality marker for the degree of their interaction/mutual solubility. A spectral-pattern similarity between the *microsponge* particles and the solid dispersions indicated the molecular-type dispersion of the former. The comparative drug-desorption study and the qualitative observations over the DSC and SEM-EDX results confirmed the successful synthesis of a homogeneous coamorphous *microsponge*-type formulation with excellent drug-loading capacity and “controlled” dissolution profile. Among them, the drug-delivery particles with 25% diltiazem content (M-25) were recognized as the most promising, with the highest population of drug molecules in the polymer bulk and the most suitable desorption profile. Furthermore, an economical and effective analytical algorithm was developed for the comprehensive physicochemical characterization of complex delivery systems of this kind.

## 1. Introduction

Acrylate and methacrylate polymers are widely used in the pharmaceutical industry as matrix- or film-forming materials [1,2]. The development of solid amorphous drug–polymer dispersions is the most common goal when subjecting them to the various techniques of molding/casting, hot-melt extrusion, 3D printing, solvent evaporation, granulation, precipitation, etc. [3,4,5]. Compared to the solid crystalline dispersions, the solid amorphous drug–polymer dispersions are characterized by increased homogeneity, permeability, drug saturation point, and drug release [2]. Not by accident, the predominantly amorphous-in-nature ammonio methacrylate copolymers (Eudragit^®^ RS and RL), butylated methacrylate copolymer (Eudragit^®^ E), and methacrylic acid–methyl methacrylate copolymer 1:1 (Eudragit^®^ L) [6] are applied as crystallization inhibitors (both on nucleation and crystal growth) in the composition of miscellaneous drug-delivery carriers [7,8,9]. The polymers’ amorphization potency depends on the processing methods and variables and the drug properties; they determine critical qualities of the final product, such as matrix/membrane permeability, porosity, stability, solubility, desorption pattern, etc. [7,10].

Ammonio methacrylate copolymer type B (Eudragit^®^ RS) is a polycationic polymer composed of methyl methacrylate and ethyl acrylate units, among which 4.5 to 7.0% methacrylic acid esters with quaternary ammonium groups are to be found [6]. The latter determine the permeability of the polymer, which indeed possesses a water-insoluble structure and pH-independent swelling [2,11,12]. Eudragit^®^ RS finds a broad application in the development of sustained-release drug-dosage forms and drug-delivery systems; it displays thermoplasticity and considerable chemical stability, wherefore is considered suitable for pharmaceutical processing [2].

**Figure 1:**
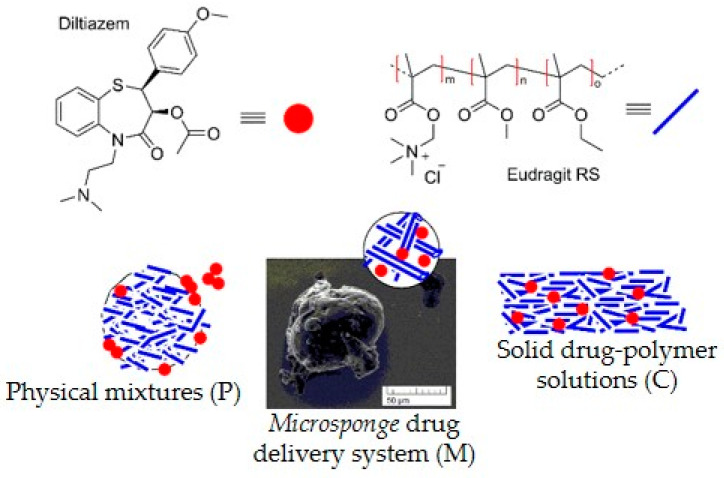
Schematic illustration of diltiazem and Eudragit RS and the expected structural assignment of their composites obtained by different techniques.

In our previous works we presented the synthesis and physicochemical, functional, and biopharmaceutical characterization of diltiazem-loaded Eudragit RS *microsponges* [13,14]–a highly preferable drug-carrier system in solid as well as in semisolid dosage forms [15,16]. The *microsponges* were designated to serve as a controlled delivery system in diltiazem 2% rectal hydrogels. The method used for their preparation (quasi-emulsion solvent diffusion method) belongs to the precipitation- and solvent-evaporation techniques [17]. To further extend the structural knowledge of the particles obtained, we herein suggest complex FTIR and sorption studies on the microporous matrix-typed *microsponges* along with two control (reference) models: physical drug–polymer mixtures and homogeneous solid drug–polymer dispersions, obtained by a casting approach (Figure 1). The morphological, elemental, and functional peculiarities of the drug-delivery system in question are additionally investigated by applying SEM-EDX and DSC.

## 2. Materials and Methods

### 2.1. Materials

Diltiazem hydrochloride 99.9% was purchased from Puho Pharmaceuticals Co. Limited, Guangzhou, China. Ammonio methacrylate copolymer (type B) (Eudragit^®^ RS 100) was a kind gift from Evonik Industries AG (Darmstadt, Germany). Poly(vinyl alcohol), Mw 49.000 Da was purchased from Sigma Aldrich, St. Louis, MI, USA. Dialysis membranes were provided from a local hospital (10 kDa molecular weight cutoff). The solvents ethanol (anhydrous) and dichloromethane (DCM, HPLC grade > 97.8%) were purchased from Sigma-Aldrich (St. Louis, MO, USA).

### 2.2. Methods

#### 2.2.1. Sample Preparation

Three working models of diltiazem-Eudragit RS mixtures were prepared, each at a minimum of three concentrations: 5%, 15%, and 25% diltiazem content. Additionally, the reference formulations (explained below) were also prepared at 10% and 20% diltiazem content.

Model I (subject of the study): Diltiazem-loaded Eudragit RS *microsponges* (M) were prepared by a quasi-emulsion solvent-diffusion method. In short, a molecular solution of diltiazem base and polymer Eudragit RS (in varying ratios; Table 1) in dichloromethane was droplet-wise added to an external aqueous phase, containing poly (vinyl alcohol) as surfactant. The resultant mixture was kept under continuous stirring until sufficient hardening of the particles and vaporization of the organic solvent. The particles were isolated by filtration and purified by multiple washing. A *microsponge*-type morphology was established (polymeric, microsized, highly porous spheres). The exact protocol for the synthesis is described elsewhere [13,14].

Model II (control model): Physical mixtures of diltiazem and Eudragit RS (P) were obtained by manually grinding predetermined amounts of diltiazem and Eudragit RS (Table 1) at room temperature (21 °C) for approximately 10 minutes. For the homogenization, an agate mortar and a pestle were used.

Model III (control model): Highly-dispersed diltiazem-Eudragit RS composites (solid solutions) (C) were obtained by dissolving 15.0 mg of diltiazem and precisely determined amounts of Eudragit RS in 5.0 mL dichloromethane-solvent, appropriate for both components. Each solution was cast into a clock glass and evaporated to dryness in a well-ventilated hood. The C-samples presented as thin, homogeneous, semitransparent films. When needed, samples for analysis from the C-series were extracted by scraping with a metal spatula.

#### 2.2.2. ATR-FTIR Spectroscopy

The attenuated total reflectance Fourier transform infrared (ATR-FTIR) spectra were recorded on a Tensor II FTIR spectrophotometer (Bruker, Germany) in the attenuated total reflection (ATR) mode, at a resolution of 0.4 cm^−1^ accumulating 32 scans. The spectra were collected at room temperature. The OPUS 8.0 software was used to autocorrect the spectral baselines.

#### 2.2.3. In Vitro Dissolution Test

Drug-dissolution studies were performed on EU pharmacopoeial dissolution apparatus type II (Dissolution Tester Model PT-DT 70, Pharmatest, Germany). Each analyte was weighed in an amount equivalent to 15.0 mg of diltiazem. P and M samples were suspended in 1.0 mL of distilled water and placed in cellulose acetate dialysis bags with a molecular cutoff of 10 kDa. The pouches thus formed were attached to the paddles of the dissolution tester using cotton threads. The C- specimens (together with the clock-glasses) were placed at the bottom of the dissolution vessel. As a dissolution medium, distilled water at a volume of 400 mL was used. All samples were allowed to swell freely for 24 h under static conditions prior to starting their dissolution testing. At the end of this stage, the losses of the target analyte (diltiazem) in the dissolution media during the swelling process were analyzed.

In order to initialize dissolution and diffusion-controlled drug release of diltiazem in the aqueous media, equimolar (to the applied drug) amounts of hydrochloric acid (concentrated) were added to each vessel. The dissolution tester was set at 25 ± 0.5 °C and a rotation speed of 30 rpm. The concentration of diltiazem hydrochloride was assessed at even time intervals for 9.0 h. After each sampling, fresh aliquots of the dissolution medium (1.0 mL) were added. Quantification was performed using an optimized and validated UV-Vis spectral method, described in previous work of ours [14].

#### 2.2.4. DSC

Differential scanning calorimetry tests were performed on *microsponge* (M-25) samples and the raw materials (diltiazem and Eudragit RS 100) by using DSC Q200 (TA Instruments, New Castle, DE, USA). Samples (~6–7 mg) were run in two modes: (1) heating-only mode from 0 to 250 °C at 10 °C/min heating rate and (2) heating–cooling–heating mode in the temperature interval from 0 to 250 °C at 10 °C/min. All DSC runs were performed under nitrogen flow (50 mL/min).

#### 2.2.5. Scanning Electron Microscope Images and EDX-Analysis

The air-dried samples (M-25 particles) were embedded in paraffin without additional dehydration steps to avoid dissolving the lipophilic components. The resulting blocks were cut into semithin sections (20 micrometers) using a conventional histological microtome (Microtome 1508A, Nanjing Oxy Technology and Trading Co., Ltd., Shanghai, China) and mounted on a graphite base for observation. The size and morphology of the polymeric particles (viz. M-25 sample) as well as the elemental analysis of the so-obtained microtome cuts were studied by scanning electron microscope (SEM, Tescan LYRA I XMU dual beam SEM/FIB system), equipped with an energy-dispersive X-ray analyzer (Bruker EDX detector Quantax 200).

## 3. Results and Discussion

Diltiazem was introduced in Eudragit RS matrices by different approaches in order to follow the changes in the polymers’ amorphization potency, permeability, solubilization capacity for diltiazem, and desorption behavior. The focus of the study was directed to the newly synthesized diltiazem-loaded Eudragit RS *microsponges*, herein marked as M-samples. Two other models were proposed as control formulations for the interpretation of the obtained results (Figure 1). The first control (reference) model, P-series, was designed to represent a heterogeneous drug–polymer dispersion, in which the drug molecules are primarily distributed on the polymeric particles’ surface. The second control model, C-series, on the contrary, aimed to achieve a maximum level of homogeneity and dispersivity between the two components; within this model, the drug is expected to be molecularly dissolved in the polymeric matrix, whereas only molecules in excess will show the tendency to crystallize on the polymeric surface.

### 3.1. ATR-FTIR Analysis

**Figure 2:**
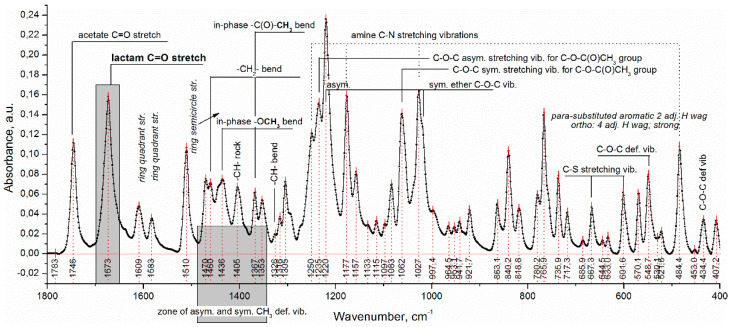
Attenuated total reflectance Fourier transform infrared (ATR-FTIR) spectrum of diltiazem base.

The complete vibrational-frequency assignments of diltiazem (base) are presented in Figure 2. Most assignments were made by comparing the spectrum of the diltiazem as obtained by us with that of its hydrochloric salt [18].

#### Carbonyl Group Vibrations

- Qualitative observations

**Figure 3:**
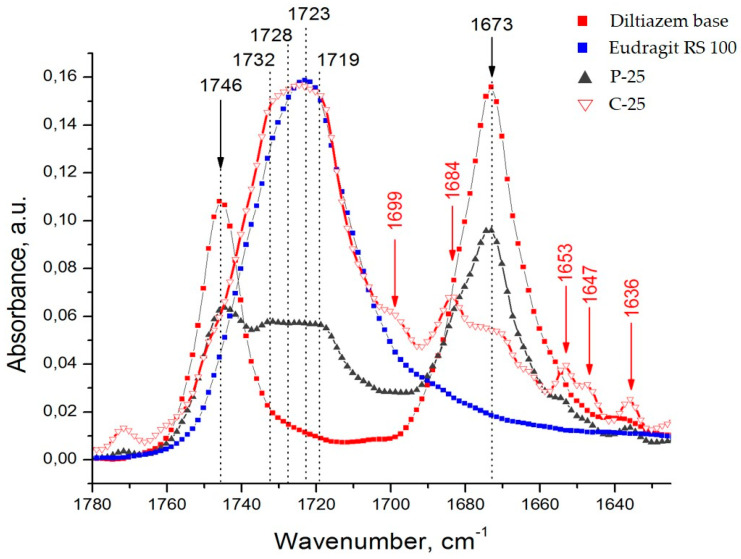
A sensitive region in the infrared spectra of diltiazem, Eudragit RS, and their control formulations—physical mixtures (P) and solid dispersions (C)—at 25% drug concentration (samples P-25 and C-25).

In the progress of our observations, we found out that the proposed ATR-FTIR method acquires satisfactory performance (with the required high analytical sensitivity and specificity) in a relatively narrow wavenumber range of about 50 cm^−1^ (from 1700 to 1650 cm^−1^) (Figure 3). Similarly, in many other circumstances, it has been assumed relevant to track out the changes in the carbonyl frequency (νCO) region when carbonyl-bearing drug molecules are examined [19,20,21].

Diltiazem shows two highly characteristic, well-defined, and readily recognizable C=O stretching bands with relatively high intensity in the range of 1760–1660 cm^−1^ (Figure 2 and Figure 3). The band observed at 1746 cm^−1^ is assigned to the CO group of the acetate functional, and that positioned at lower frequencies (with a maximum at 1673 cm^–1^) is assigned to the CO functional group of the seven-membered lactam ring [22]. In the same spectral interval, however, the presence of a very intense and broad IR band (with a max. at 1723 cm^−1^) in the spectrum of the polymer Eudragit RS 100 was also registered (Figure 3). Certainly, this last-mentioned absorption band should be considered as consisting of at least three overlapping bands corresponding to the three different kinds of grafted carbonyl (ester) groups on the polymeric backbone (Figure 1). According to the generally accepted theory of infrared spectroscopy, however, when solvent–solute mixtures of two or more constituents are examined (as in the case here studied), considerable frequency shifts of all carbonyl groups due to mutual interference effects are expected to appear. Usually, the used solvent (a liquid of low-molecular-weight organic substance) or a high-molecular organic matrix (polymer) affects both the position and the intensity of each absorption band of the examined solute (diltiazem) via the so-called solvent effect. Indeed, when the spectra of diltiazem–Eudragit RS physical mixtures (P) and solid solutions (C) were analyzed, considerable differences in the CO frequency pattern of each participant from its native spectrum were registered (Figure 3).

Concerning the lower-frequency subzone, from 1690 to 1660 cm^−1^ (Figure 3), the appearance of a new absorption band at 1684 cm^−1^ in the spectra of both types of mixtures (P and C models) was recorded. In the case of the physical mixtures, the presence of this band (normally detected as a shoulder on the high-energy tail of the primary band at 1673 cm^−1^) was, as a whole, less pronounced. In the spectra of the solid solutions (C), however, this band occurs as spectrally predominant and thus indicative of the successful dispersing of drug molecules in the polymer matrix. It is reasonable to assume that the emerging new band increases its intensity as a function of the extent of mixing (dispersing) the drug molecules into the polymeric bulk, whereas the lower one, at 1673 cm^−1^, accordingly decreases its integral transparency. The shift in the observed CO frequency of about 11 cm^−1^ should, in principle, be attributed to the effect of strong dipole–dipole interaction between molecules of the used solvent, Eudragit RS 100, and the dispersed solute, diltiazem.

- Quantitative observations

**Figure 4:**
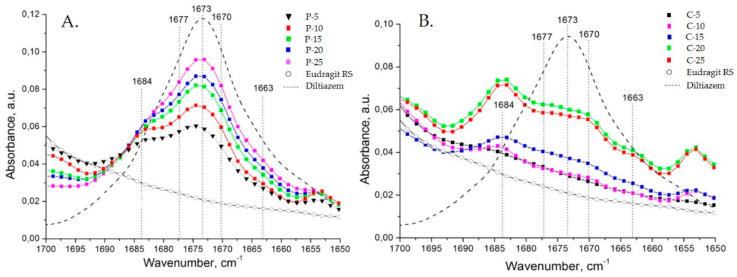
Concentration-dependent changes in the infrared spectra of (**A**) physical diltiazem-Eudragit RS mixtures (P) and (**B**) solid diltiazem–Eudragit RS solutions (highly dispersed composites) (C).

All types of diltiazem–Eudragit RS mixtures were studied at varying concentrations in order to determine the point of saturation for each of the techniques applied and follow other concentration-dependent changes within the polymer matrix (Figure 4).

**Figure 5:**
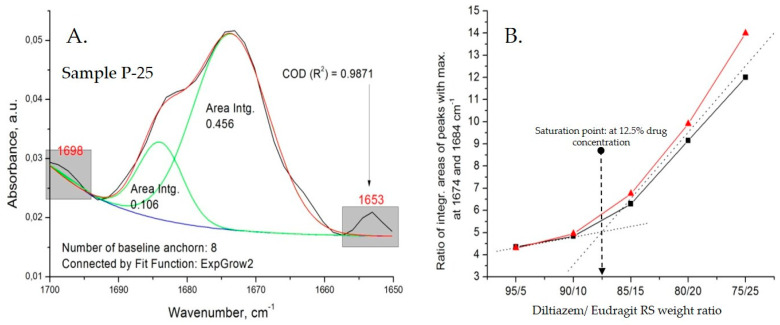
Deconvolution analysis in the C=O (lactamic) stretching vibrations range for physical drug–polymer mixtures (P models); (**A**) an example for deconvolution analysis carried on Sample P-25 spectrum (the same analysis is carried for all other samples-Table A1, Table A2, Table A3, Table A4, Table A5, Table A6, Table A7, Table A8, Table A9 and Table A10, Appendix A) and (**B**) determination of drug-saturation point of the polymer matrix by the physical-mixing approach; the curve in red presents optimized processing of the deconvolution analysis data, in which the cause for the low regression coefficient (R^2^)–a peak at 1653 cm^−1^, is not taken into account.

The appearance of a C=O stretching vibration band at 1684 cm^−1^ (as a “shoulder” to the intrinsic 1673 cm^−1^ band) in the spectra of all diltiazem–Eudragit RS 100 physical mixtures (P) should be directly ascribed to the particularities of the preparation technique. Upon mixing of the two constituents, due to significant variations in their hardness and the relatively high coefficient of applied friction, an effect of partial mutual diffusion (penetration) between both ground constituents is expected to occur at the points of contact. Thus, the “distinct” neighboring molecules diffuse, creating a heterogeneous composite mixture of microparticles consisting of a polymeric core and an outer “functionalized” shell of irregularly organized diltiazem molecules. As the drug content increases, however, the population of diltiazem molecules included in the polymeric volume reaches its limit (the point of saturation), while the molecules in excess form an intact diltiazem phase; the latter displays the characteristic for the drug IR absorption behavior (i.e., abs. maximum at 1673 cm^−1^). Approaching the drug-saturation point of the polymeric surface, no further increase in the intensity of the 1684 cm^−1^ band is observed, whereas the 1673 cm^−1^ band continues to “grow”. A deconvolution analysis in the C=O (lactamic) stretching vibrations range (from 1700 to 1650 cm^−1^), performed with the aid of specialized peak-fitting software, Origin 8.1 (Table A1, Table A2, Table A3, Table A4, Table A5, Table A6, Table A7, Table A8, Table A9 and Table A10, Appendix A), allowed us to define the exact point of polymer saturation with diltiazem when the drug is introduced via mechanical grinding and mixing (P models) (Figure 5).

**Figure 6:**
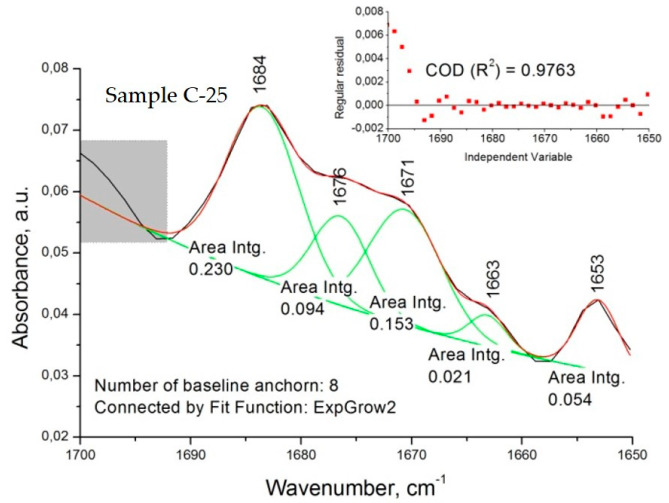
An example of deconvolution analysis carried out on Sample C-25 spectrum.

Concerning the spectra of diltiazem–Eudragit RS solid solutions (C models), significant changes in the considered wavenumber range were registered. The appearance of several new bands was observed for all concentrations examined (Figure 4B and Figure 6). Each of the newly appearing bands may be assigned to a complex intermolecular interaction between the two constituents, diltiazem and Eudragit RS. The good mutual solubility between them was illustrated and confirmed by the appearance of three new bands in the C=O stretching region (at 1663, 1670/1671, and 1684 cm^−1^) and by the reduction in the intensity of the absorption band at 1673 cm^−1^. Nonetheless, the spectral patterns of the highly dispersed mixtures (C probes) were considered too complex for a clear identification to be made. Still, it is reasonable to assume that during the process of homogeneous dispersion, the drug molecules occupy regions of the polymeric matrix with different degree of crystallinity, and to a certain extent dielectricity, which produce detectable frequency variations in νCO of the drug solute.

**Figure 7:**
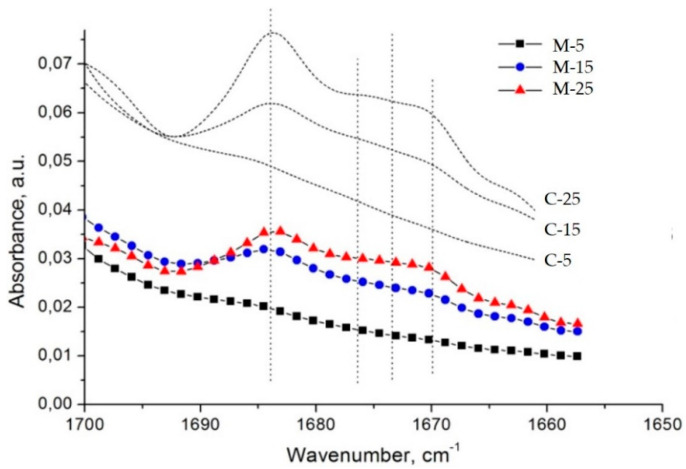
Spectral similarity between the infrared spectra of drug-loaded *microsponges* (M) and drug–polymer solid dispersions (C).

Strangely enough, the FTIR spectra of the *microsponge* samples (M samples) imitate the spectral profile of the solid drug–polymer solutions (C samples) (Figure 7). This observation does not correspond to the fact that—as shown in Figure 7—the population of dispersed diltiazem molecules (absorbing at 1684 cm^−1^) in the bulk of the two kinds of materials is different. Comparing the respective peak heights of the relevant bands in the spectrum of the M-25 sample, one could see that the proportion of occluded to easily diffusing (absorbing at 1673 cm^−1^) diltiazem molecules appears to be significantly lower than that of its C-25 equivalent. As it will be shown below, these observations have led us to an adequate explanation of the unusual desorption behavior of the sample in question at neutral pH. However, such a result testifies for good homogeneity and amorphization of diltiazem within the *microsponge*-type polymeric matrix.

### 3.2. Dissolution Rate Study

**Figure 8:**
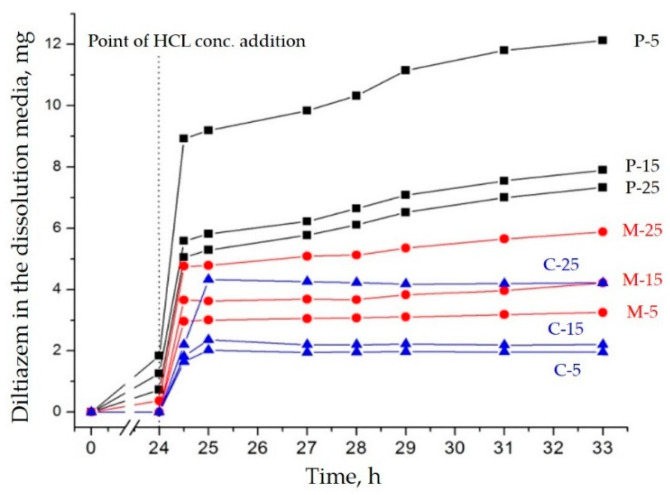
Diltiazem release from physical mixtures (P), solid dispersions (C), and *microsponge* (M) formulations.

All diltiazem–Eudragit RS models were subjected to drug dissolution (desorption) studies in order to relate their structural features to some important functional properties: apparent solubility, drug diffusivity, and matrix permeability. To identify the potential resultant differences as a function of the diffusion-controlled drug release in particular, all samples were allowed to swell before initializing the drug dissolution. The period of swelling (24 h) slightly affected the drug load in the matrices since diltiazem base is a strictly hydrophobic molecule [13]. Only when a concentrated solution of hydrochloric acid was applied in a stoichiometrically calculated volume ratio (after the “swelling” stage) was the drug base allowed to form its highly water-soluble salt (453 mg/mL in distilled water at 37 °C [13]) and diffuse into the aqueous media (Figure 8).

The physical drug–polymer mixtures (P) showed the fastest and most complete drug-release rate among all models. This result supports the data from the FTIR analysis, which indicated a low degree of interaction between the components upon their mixing by mechanical grinding. As the drug is only superficially located onto the polymeric particles, there are not expected to be any particular barriers to its diffusion, except its intrinsic solubility. Moreover, as the relative drug content in the P-models increases, the dissolution rate decreases. Eudragit RS may be here reviewed as a “solid” mediator for the drug particles’ size reduction; therefore, the higher the polymeric content in these mixtures, the larger the active surface area of diltiazem and the faster the dissolution.

The casting-obtained C-probes demonstrated minimal and incomplete drug desorption. Based on the FTIR results and the visual appearance of these samples (semitransparent films), it could be claimed that this model provides less potency for drug/polymer amorphization and entraps diltiazem in a much denser, less permeable, and more organized polymeric matrix. As solid solutions are formed, the drug excess, however, showed the tendency to crystalize on the polymeric surface. Accordingly, the higher the relative drug content, the higher the excess and the released drug portion.

The sorption behavior of the *microsponge* samples (M) showed an intermediate position compared to the two reference models (P and C). However, a resemblance was observed with the C formulations. The polymeric matrix possesses a higher capacity for diltiazem incorporation and greater permeability when a porous structure is obtained (as in the case of the drug-loaded microsponges [13]). In this regard, the M samples, although proven to be as highly dispersed as the C samples, ensure faster and more complete drug release after matrix swelling. The more significant the polymeric contribution within the *microsponge* particles, the longer the diffusion path for diltiazem; hence, samples with higher relative drug content and respective lower polymeric content showed superiority with respect to drug release.

### 3.3. DSC Analysis

**Figure 9:**
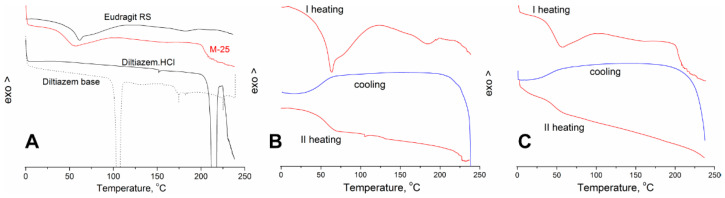
(**A**) Thermograms of diltiazem base, diltiazem hydrochloride, Eudragit RS 100, and M-25 sample in heating-only mode; (**B**) thermogram in the heating–cooling–heating mode of Eudragit RS 100; (**C**) thermogram in the heating–cooling–heating mode of M-25 sample.

Figure 9A shows the thermograms obtained via the heating-only mode for diltiazem (base), diltiazem hydrochloride, Eudragit RS 100, and M-25 sample. Diltiazem base was demonstrated to melt at 103.8 °C, which is in agreement with the literature data (105–107 °C) [23]. From the same figure, we detected a melting point at 214.5 °C for the hydrochloride salt, which again corresponds well to the literature [23]. The melting of diltiazem hydrochloride (~225 °C) is accompanied by decomposition, which becomes apparent above 230 °C [18]. Two peaks, at 63.1 and 183.6 °C, appear in the thermogram of the pure Eudragit RS 100 polymer. The first is the glass transition, which is reported to occur between 61.9 and 64.9 °C [24]. The high temperature peak (183.6 °C) is due to Eudragit RS 100 melting, which is known to occur at ~187 °C [25].

In the thermogram of the M-25 sample, the melting peak of diltiazem disappears. This result confirms that the drug is in the amorphous state and that the formulation is, in fact, a molecular dispersion of diltiazem inside the polymer [26]. Similar findings are characteristic of highly dispersed systems (solid solutions) obtained via hot-melt extrusion, for example [25,27]. The interaction between the drug and the polymer results in the amorphization of both constituents within the M-25 formulation, hence the lack of their respective melting peaks. Another observation that could be seen is the appearance of a new peak at a temperature just above 200 °C. A possible explanation is the in situ formation of diltiazem hydrochloride salt (melting point at ~225 °C), as diltiazem base in the particles is prone to react with the polymeric [Cl^−^] counterions (Figure 1). Such ion-exchange reactions are reported for Eudragit RS upon melting [28]. Another reasonable explanation could be the degradation of diltiazem (detected for the pure drug above 230 °C) at a lower temperature due to its amorphization and molecular dispersivity in the polymer matrix. The decrease in the polymer’s glass transition in the M-25 composition by 13 °C (as compared to that of the pure Eudragit RS) is likely due to the formation of a looser polymer network and increased free volume in the presence of diltiazem [29].

The heating–cooling–heating mode results (Figure 9B,C) confirm the abovementioned observations. After cooling and second heating, the glass transition of Eudragit RS (63.1 °C) manifests without the relaxation enthalpy peak, and a kink typical for an irreversible process appears.

### 3.4. SEM-EDX Analysis

**Figure 10:**
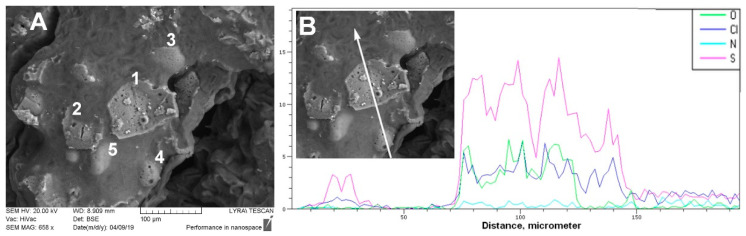
(**A**) SEM micrograph on microtome-made cuts of *microsponge* particles M-25 and (**B**) EDX analysis.

SEM-EDX technique was applied to identify the elemental composition along a cut made on paraffin-embedded particles from the M-25 formulation. The primary morphological characteristics are shown in Figure 10A. On the micrograph obtained, we distinguished and marked three zones, namely (1) clear cuts/slices of *microsponge* particles (#1 and #2); (2) intact *microsponge* particles (#3, #4, and #5); and (3) paraffin base (surrounding the visible particles). The white arrow on Figure 10B shows exactly where an EDX scan analysis was performed. In the same figure, the dynamics of the elemental composition (for S, N, O, and Cl) along the cut can be followed.

Our attention was directed to the sulfur (S), which indicates the distribution of diltiazem (S-containing drug) in the polymeric matrix-type particles. As no specific cumulation patterns were recognized on the scan, we should conclude that the drug possesses a relatively even distribution in the polymeric volume. The observed fluctuation in the S content, as well as in the other elements’ content, could be attributed to the architectural peculiarities of this type of *microsponge* materials, consisting of randomly alternating zones of hollow micropores (in which the X-ray emission is reduced) and solid walls.

## 4. Conclusions

Combining some of the benefits of infrared spectrophotometry, differential scanning calorimetry, energy-dispersive X-ray microanalysis, and dissolution testing, we proposed a methodological algorithm by which we were able to analyze the molecular distribution of diltiazem in the bulk of Eudragit RS 100-based particles of microspongeous type. For the purposes of the analysis, we proved the ability to accurately relate the spectral and desorption behavior of our drug-delivery systems to analogous reference formulations—physical mixtures and highly dispersed composites (solid dispersions). Based on the methodology set out, we were able to establish that the sample preparation technique for this type of drug carriers results in an even, homogeneous, and molecular-type drug distribution within the polymer matrix. Despite the spectral similarity with the solid drug–polymer solutions, the drug-delivery particles presented a substantially increased drug-dissolution rate owing to their porosity. For the sake of comparison, the microsponge model was proved to combine the benefits of both reference models, namely molecular distribution in the polymeric bulk (needed to exert control over the drug release) and satisfactory drug-solubility rate. The result is considered highly valuable for the development of controlled-release drug-delivery systems. In addition, this work presents an economical and rapid algorithm for the correct analysis of drug carriers of this type.

## Figures and Tables

**Table 1:** Mass ratios of diltiazem and the polymer Eudragit RS 100.

Code	Used Amounts of Diltiazem and Eudragit RS, W/W%
P-5	5/95
P-10	10/90
P-15	15/85
P-20	20/80
P-25	25/75
C-5	5/95
C-10	10/90
C-15	15/85
C-20	20/80
C-25	25/75
M-5	5/95
M-15	15/85
M-25	25/75

## Data Availability

Not applicable.

## Appendix A

Spectral deconvolution is an algorithm-based curve-fitting process used to find the best collection of overlapped peaks; whose sum closely matches their composite (original) spectrum.

The integral areas of individual peaks were determined using the curve-fitting application of Origin 8.1 software. All spectra were deconvoluted in the range of C=O (lactamic) stretching vibrations (from 1700 to 1650 cm^−1^). To obtain reliable results, a rational choice of several parameters was also applied. Validation tests based on analysis of variance (ANOVA) were also accomplished. The results of the analyses are given in the following tables:

**Table A1:** Sample P-5.

	**STATISTICS**		**ANOVA**						
		B			DF	Sum of squares	Mean square	F value	Prob. > F
	Number of points	36	B	Regression	6	0.0648	0.0108	11,282.11	0
	Degrees of freedom	30		Residual	30	2.8718 × 10^−5^	9.5725 × 10^−7^		
	Reduced Chi-square	9.5725 × 10^−7^		Uncorrected total	36	0.06483			
	Adj. R-square	0.99522		Corrected total	35	0.00701			
	Fit status	Succeeded (100)							

**Table A2:** Sample P-10.

	**STATISTICS**		**ANOVA**						
		B			DF	Sum of squares	Mean square	F value	Prob. > F
	Number of points	36	B	Regression	6	0.06965	0.01161	65.297	3.21 × 10^−15^
	Degrees of freedom	30		Residual	30	0.00533	1.778 × 10^−4^		
	Reduced Chi-square	1.778 × 10^−4^		Uncorrected total	36	0.07499			
	Adj. R-square	0.9729		Corrected total	35	0.2298			
	Fit status	Succeeded (100)							

**Table A3:** Sample P-15.

	**STATISTICS**		**ANOVA**						
		B			DF	Sum of squares	Mean square	F value	Prob. > F
	Number of points	36	B	Regression	6	0.08577	0.01429	5621.55	0
	Degrees of freedom	30		Residual	30	7.62844 × 10^−5^	2.54281 × 10^−6^		
	Reduced Chi-square	2.54281 × 10^−6^		Uncorrected total	36	0.08584			
	Adj. R-square	0.9934		Corrected total	35	0.01342			
	Fit status	Succeeded (100)							

**Table A4:** Sample P-20.

	**STATISTICS**		**ANOVA**						
		B			DF	Sum of squares	Mean square	F value	Prob. > F
	Number of points	36	B	Regression	6	0.09502	0.01584	3720.66	0
	Degrees of freedom	30		Residual	30	1.277 × 10^−4^	4.256 × 10^−6^		
	Reduced Chi-square	4.256 × 10^−6^		Uncorrected total	36	0.09515			
	Adj. R-square	0.9906		Corrected total	35	0.01577			
	Fit status	Succeeded (100)							

**Table A5:** Sample P-25.

	**STATISTICS**		**ANOVA**						
		B			DF	Sum of squares	Mean square	F value	Prob. > F
	Number of points	36	B	Regression	6	0.10619	0.0177	2531.19	0
	Degrees of freedom	30		Residual	30	2.09757 × 10^−4^	6.99189 × 10^−6^		
	Reduced Chi-square	6.99189 × 10^−6^		Uncorrected total	36	0.1064			
	Adj. R-square	0.9882		Corrected total	35	0.02074			
	Fit status	Succeeded (100)							

**Table A6:** Sample P-5 rep.

	**STATISTICS**		**ANOVA**						
		B			DF	Sum of squares	Mean square	F value	Prob. > F
	Number of points	36	B	Regression	8	0.0387	0.00646	3615.75	0
	Degrees of freedom	30		Residual	28	5.36 × 10^−5^	1.79 × 10^−6^		
	Reduced Chi-square	1.786 × 10^−6^		Uncorrected total	36	0.0388			
	Adj. R-square	0.98493		Corrected total	35	0.00415			
	Fit status	Succeeded (101)							

**Table A7:** Sample P-10 rep.

	**STATISTICS**		**ANOVA**						
		B			DF	Sum of squares	Mean square	F value	Prob. > F
	Number of points	36	B	Regression	8	0.0347	0.00434	10,165.88	0
	Degrees of freedom	28		Residual	28	1.195 × 10^−4^	4.269 × 10^−6^		
	Reduced Chi-square	4.269 × 10^−6^		Uncorrected total	36	0.03473			
	Adj. R-square	0.99762		Corrected total	35	0.00628			
	Fit status	Succeeded (101)							

**Table A8:** Sample P-15 rep.

	**STATISTICS**		**ANOVA**						
		B			DF	Sum of squares	Mean square	F value	Prob. > F
	Number of points	36	B	Regression	6	0.0801	0.01335	5495.559	0
	Degrees of freedom	30		Residual	30	7.287 × 10^−5^	2.429 × 10^−6^		
	Reduced Chi-square	2.429 × 10^−6^		Uncorrected total	36	0.08017			
	Adj. R-square	0.99376		Corrected total	35	0.01362			
	Fit status	Succeeded (100)							

**Table A9:** Sample P-20 rep.

	**STATISTICS**		**ANOVA**						
		B			DF	Sum of squares	Mean square	F value	Prob. > F
	Number of points	36	B	Regression	8	0.08498	0.01062	7789.934	0
	Degrees of freedom	28		Residual	28	3.818 × 10^−5^	1.3636 × 10^−6^		
	Reduced Chi-Square	1.3636 × 10^−6^		Uncorrected total	36	0.08502			
	Adj. R-square	0.99777		Corrected total	35	0.02142			
	Fit status	Succeeded (100)							

**Table A10:** Sample P-25 rep.

	**STATISTICS**		**ANOVA**						
		B			DF	Sum of squares	Mean square	F value	Prob. > F
	Number of points	36	B	Regression	8	0.06424	0.00803	5404.575	0
	Degrees of freedom	28		Residual	28	4.16018 × 10^−5^	1.4858 × 10^−6^		
	Reduced Chi-square	1.4858 × 10^−6^		Uncorrected total	36	0.06428			
	Adj. R-square	0.99673		Corrected total	35	0.0159			
	Fit status	Succeeded (100)							
